# *In vitro* generation of RORγt^+^ regulatory T cells reveals enhanced immunosuppressive function and OXPHOS-dependent metabolism

**DOI:** 10.3389/fimmu.2026.1742866

**Published:** 2026-05-21

**Authors:** Marcella Cipelli, Eloísa Martins da Silva, Lais Cavalieri Paredes, Mariana Abrantes do Amaral, Barbara Nunes Padovani, Luísa Menezes-Silva, Vinicius Andrade-Oliveira, Niels Olsen Saraiva Camara

**Affiliations:** 1 Department of Immunology, Institute of Biomedical Sciences, University of São Paulo, São Paulo, SP, Brazil; 2 Department of Medicine, Discipline of Nephrology, Federal University of São Paulo, São Paulo, SP, Brazil; 3 Center for Human and Natural Sciences, Federal University of ABC, São Paulo, SP, Brazil

**Keywords:** immunometabolism, RORγt, T cell differentiation, T cell function, Treg

## Abstract

**Background:**

RORγt^+^ regulatory T cells (Treg) play a crucial role in immune regulation, particularly in the gut. However, most current knowledge about this subset derives from *in vivo* studies, as *in vitro* investigation has been limited by the lack of protocols capable of preserving their phenotype.

**Methods:**

Here, we developed and optimized an *in vitro* differentiation protocol to efficiently generate RORγt^+^ Treg cells. The protocol was evaluated based on the frequency of RORγt^+^ Treg cells generated, their suppressive function compared to conventional induced Treg (iTreg), and their metabolic profile.

**Results:**

The optimized protocol increased the frequency of RORγt^+^ Treg cells *in vitro* by up to 70%, providing a robust system for their study. Functionally, *in vitro*–differentiated RORγt^+^ Treg cells displayed enhanced immunosuppressive activity compared to conventional iTreg, effectively inhibiting effector CD4⁺ T cell proliferation. Metabolic analyses further revealed a reliance on oxidative phosphorylation (OXPHOS) in this subset.

**Conclusion:**

This protocol enables the efficient *in vitro* generation of RORγt^+^ Treg cells, facilitating functional and metabolic studies of this population and opening new avenues for potential therapeutic applications in immune-mediated diseases.

## 1 Introduction

Regulatory T cells (Treg) play a central role in maintaining immune homeostasis by controlling inflammation and promoting tolerance to self-antigens and the microbiota. Among Treg subtypes, those expressing the transcription factor RORγt (RORγt^+^ Treg) are particularly important in directing intestinal immunity. These cells exhibit a hybrid phenotype, combining immunosuppressive properties of conventional Treg—characterized by FOXP3 expression and suppression of effector T cell responses—with certain functional attributes of Th17 cells, such as RORγt expression and the ability to produce IL-17 (1, 2). However, evidence suggests that RORγt^+^ Treg are essential for suppressing excessive immune activation in the gut, helping to prevent inflammatory bowel diseases (IBD) and other microbiota-related disorders (3, 4). *In vivo* differentiation of RORγt^+^ Treg cells is controlled by multiple factors in the intestinal microenvironment, including cytokines and tissue-derived signals. Notably, IL-6 influences both the rate and functional profile of these cells (5). The transcription factor c-Maf has been identified as an important regulator for Treg specialization into the RORγt^+^ phenotype. This indicates that their functional specialization is governed by distinct transcriptional programs (6).

Despite growing knowledge of the biology of RORγt^+^ Treg *in vivo*, reliable protocols for their *in vitro* differentiation remain inconsistent. Some studies have successfully polarized these cells from naïve T cells by applying specific cytokine combinations, such as TGF-β and IL-6, along with additional factors, such as retinoic acid (7). However, substantial variability exists among published protocols regarding cytokine composition, timing of stimulation, and culture conditions, frequently resulting in marked differences in differentiation efficiency, Foxp3 and RORγt co-expression stability, and contamination with Th17-like populations (1, 8).

This methodological aspect of heterogeneity represents a major limitation to reproducibility across the literature and hampers mechanistic comparisons and translational efforts involving RORγt^+^ Treg cells. Establishing a standardized, optimized protocol for the *in vitro* generation of RORγt^+^ Treg cells would substantially advance research in this field. An effective differentiation method would enable a greater understanding of the elements governing their development and function, while assisting in their therapeutic use for inflammatory and autoimmune diseases. In this study, we present a protocol for the induction of RORγt^+^ Treg in culture, refining conditions to improve their steadiness and immunoregulatory properties.

## 2 Materials and methods

### 2.1 Mice

Male C57BL/6 mice, aged 8 to 10 weeks and weighing between 18 and 25 g, were used for the experiments. Male mice from the Foxp3GFP RORγtTomato lineage were also used under the same conditions as the C57BL/6 strain. To generate this lineage, mice with the fluorescent GFP protein inserted into the Foxp3 gene (Foxp3GFP knock-in, The Jackson Laboratories) were crossed with mice carrying the tdTomatoflox/flox allele over several generations until they produced homozygous (or hemizygous, in the case of the Foxp3 gene in males) offspring for both genes (Foxp3GFP+/+ tdTomatoflox/flox). These offspring were then crossed with mice carrying the Cre enzyme in the Rorc gene, allowing the expression of the fluorescently labeled tdTomato protein specifically in RORγt cells by removing the “stop coding” via the Cre-Lox system. We used the nomenclature Foxp3GFPRORγtTomato for the double-reporter animals. The genotyping of the three genes was performed by conventional PCR. All strains were maintained under specific pathogen-free conditions, with 12-hour light/dark cycles and sterile food and water provided *ad libitum*. The experiments were conducted in accordance with local regulations governing the use of animals in scientific studies and were approved by the Ethics Committee for Research in the Institute of Biomedical Sciences at the University of São Paulo (Process No 7288101218).

### 2.2 Differentiation of RORγt^+^ regulatory T cells *in vitro*

Naïve CD4^+^ T cells were isolated from spleen and peripheral lymph nodes of 5–10-week-old C57BL/6 mice or Foxp3GFPRORγtTomato reporter mice by cell sorting or using a negative selection strategy with the EasySep Mouse Naïve CD4^+^ T Cell Isolation Kit (StemCell Technologies), according to the manufacturer’s instructions. Cells were cultured in complete RPMI 1640 medium supplemented with 10% heat-inactivated fetal bovine serum, 2 mM L-glutamine, 100 U/mL penicillin, 100 µg/mL streptomycin, and 50 µM β-mercaptoethanol (Sigma-Aldrich). For the initial differentiation of induced regulatory T cells (iTreg), flat-bottom 96-well plates were pre-coated with anti-CD3ϵ antibody (clone 145-2C11, BioLegend) at a concentration of 2 µg/mL diluted in PBS (50 µL per well) and incubated for 4 h at 37 °C in a humidified incubator with 5% CO_2_. After coating, the plates were washed three times with PBS at room temperature. Naïve CD4^+^ T cells were then seeded at a density of 2 × 10^5^ cells per well in 100 µL of complete RPMI. Cells were stimulated by the addition of 100 µL per well of a 2× concentrated iTreg polarization cocktail containing soluble anti-CD28 antibody (clone 37.51, 1 µg/mL), neutralizing antibodies against IL-4 (clone 11B11, 1 µg/mL), IFN-γ (clone XMG1.2, 1 µg/mL), and IL-12/IL-23 p40 (clone C17.8, 1 µg/mL), recombinant murine IL-2 (100 U/mL), and recombinant murine TGF-β (5 ng/mL). Cultures were maintained for 4 days at 37 °C with 5% CO_2_ to allow efficient Foxp3^+^ iTreg differentiation.

To induce the generation of double-positive Foxp3^+^RORγt^+^ Treg, cells differentiated under iTreg conditions were harvested on day 4, pooled, counted, and resuspended in fresh complete medium. A new 96-well flat-bottom plate was pre-coated with anti-CD3ϵ (2 µg/mL) under the same conditions described above. Cells were then re-seeded at a density of 2 × 10^5^ cells per well and re-stimulated with anti-CD28 (1 µg/mL) in the presence of a modified polarization cocktail designed to promote RORγt expression while continuing to maintain Foxp3 stability. This second-phase cocktail contained neutralizing antibodies against IL-4 (1 µg/mL) and IFN-γ (1 µg/mL), recombinant murine IL-6 at concentrations ranging from 2.5 to 5 ng/mL (or dose–response curves when indicated), recombinant murine IL-2 at a reduced concentration (50 U/mL), and recombinant murine TGF-β (5 ng/mL). Cells were cultured under these conditions for an additional 4 days. Detailed data of catalog numbers from cytokines and neutralizing antibodies are shown in **Supplementary Table 1**.

At the end of the differentiation protocol (day 8), cells were collected and stained for viability using a Live/Dead Cell Viability Assay (Invitrogen), followed by surface staining with anti-CD4 antibody (clone GK1.5), anti-CTLA-4 antibody (clone UC10-4F10-11), and anti-PD-1 antibody (clone J43). Intracellular staining was performed after fixation and permeabilization using antibodies against Foxp3 (clone MF-14) and RORγt (clone Q31-378). See a detailed staining panel in **Supplementary Table 2**. Data acquisition was performed on a FACSCanto II flow cytometer using FACSDiva software (BD Biosciences), and data were subsequently analyzed using FlowJo software (Tree Star, San Carlos, CA, USA). Detailed information about antibodies is shown in **Supplementary Table 2**.

### 2.3 *In vitro* Treg suppression assay

Total splenocytes were labeled with CellTrace Violet reagent (Invitrogen) according to the manufacturer’s instructions and plated at 75 × 10³ cells per well in round-bottom 96-well plates. *In vitro*–differentiated RORγt^+^ Treg and iTreg from Foxp3GFPRORγtTomato mice were purified prior to the assay by fluorescence-activated cell sorting (FACS) based on the expression of CD4, Foxp3, and RORγt. Post-sort purity was routinely assessed by flow cytometry and exceeded 98% for all experiments. Purified RORγt^+^ Treg were co-cultured with CellTrace Violet–labeled responder splenocytes at Treg-to-responder ratios of 2:1 (150 × 10³ cells/well), 1:1, 1:2, 1:4, 1:8, and 1:16. T cell proliferation was stimulated with soluble anti-CD3 antibody (1 μg/mL). As a suppression control, *in vitro*–generated conventional induced Treg (iTreg) were included at identical ratios. Cells were maintained in complete RPMI medium for 72 h at 37 °C with 5% CO_2_ in a humidified incubator. After incubation, cells were harvested and stained with a Live/Dead viability dye (Life Technologies) and antibodies against CD4 (clone GK1.5) and CD8 (clone 53-6.7). Proliferation of responder T cells was quantified by flow cytometric analysis of CellTrace Violet dilution within viable CD4^+^ and CD8^+^ T cell gates (detailed information about antibodies is shown in **Supplementary Table 3**). Suppression was calculated as the percentage reduction in proliferation relative to cultures stimulated with anti-CD3 in the absence of regulatory T cells.

### 2.4 Seahorse real-time cell metabolic analysis

Oxygen consumption rates (OCR) were quantified using an XF-96 extracellular flux analyzer (XF-96 Extracellular Flux Analyzer - Seahorse, Agilent) to obtain knowledge about mitochondrial respiration. For this, Treg, Th17, and RORγt+ Treg were differentiated *in vitro* and plated at 400,000 cells per well in an XF-96 plate, which had been treated with 100 mg/mL Poly-D-Lysine (Sigma-Aldrich) for 2 hours at room temperature, then washed with PBS 1x twice. RPMI 1640 culture medium (Gibco) without buffering, supplemented with 2 mM glutamine (Sigma Aldrich), 25 mM glucose (Sigma Aldrich), and 1 mM sodium pyruvate (Sigma Aldrich), was used for cell maintenance. During the assay, Oligomycin (1 μg/mL, ATP synthase inhibitor), CCCP (5 μM, Carbonyl cyanide-4 (trifluoromethoxy) phenylhydrazone, a potent uncoupler and mitochondrial respiratory chain accelerator), and Antimycin-A (1 μM)/Rotenone (1 μg/mL, both electron transport chain inhibitors) were injected each in the pre-defined timing for MitoStress Test. The mitochondrial parameters analyzed included basal respiration, ATP-linked respiration, maximal respiration, spare respiratory capacity, and non-mitochondrial respiration. Extracellular acidification rates (ECAR) were also quantified using the XF-96 extracellular flux analyzer (XF-96 Extracellular Flux Analyzer - Seahorse, Agilent). During the assay, Glucose (25 mM), Oligomycin (1 μg/mL, an ATP synthase inhibitor), and 2-DG (2-deoxy-D-glucose at 20 mM) were injected at the predefined times for the Glycolysis Stress Test, providing data on glycolytic pathway activation. Glycolytic parameters included basal glycolysis, glycolytic capacity, and glycolytic reserve. OCR and ECAR values were normalized to cell number per well.

### 2.5 Enzyme-linked immunosorbent assay

Enzyme-linked immunosorbent assays were used to quantify IL-6 and IL-10 from the cell culture supernatants. Kits were purchased from R&D Systems (Minneapolis, MN, USA) and performed as described by the manufacturers.

### 2.6 Statistical analysis

Statistical analysis was performed using GraphPad Prism^®^ software (San Diego, CA, USA). Differences among groups were compared using Student’s t-test, one-way or two-way ANOVA with Bonferroni post-test, as indicated. Based on the Kolmogorov-Smirnov test for normality, we selected the Pearson or Spearman test for gene correlation analysis, as indicated in the respective figure. The observed differences were considered significant when p < 0.05 (5%).

## 3 Results

### 3.1 The combination of IL-6 and TGF-β after a second stimulus in iTreg leads to the acquisition of RORγt

**Figure 1:**
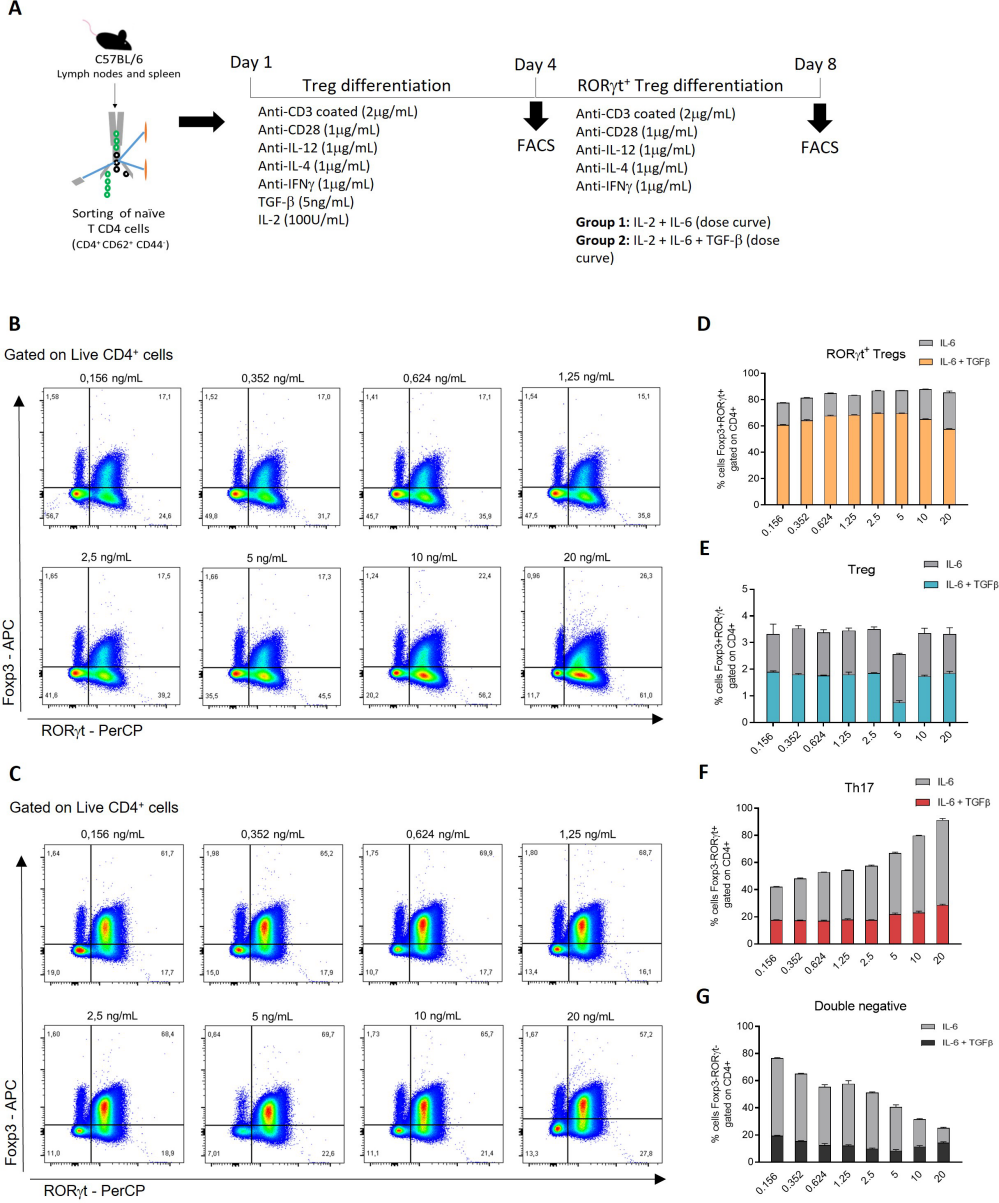
Establishing an optimized protocol for RORγt^+^ Treg differentiation *in vitro*. **(A)** overview of RORγt^+^ Treg *in vitro* differentiation protocol. **(B, C)** naïve CD4+ T cells were differentiated into Treg *in vitro*, and on the fourth day of the experiment, they were cultured in a new culture plate containing anti-CD3 bound, anti-CD28, IL-6, and IL-2 or IL-2 + TGF-β. The IL-6 concentrations used are indicated above the graphs. After the eighth day of the experiment, the cells were stained with live/dead viability dye, anti-CD4, anti-Foxp3, and anti-RORγt. Representative gating demonstrates the frequency (%) of Foxp3+; RORγt+ and Foxp3+; RORγt- after the selection of singlets/Live/Dead^-^ and CD4 +. **(D-G)** frequencies of populations after *in vitro* differentiation. Data refer to the percentages of RORγt^+^ Treg (live CD4^+^Foxp3^+^RORγt^+^), Treg (live CD4^+^Foxp3^+^RORγt^-^), Th17 cells (live CD4^+^Foxp3^-^RORγt^+^) and double negative cells (live CD4^+^Foxp3^-^RORγt^-^), obtained from a pool of mice (n=4).

To establish an *in vitro* protocol for the generation of RORγt^+^ Treg (Foxp3^+^RORγt^+^), naïve CD4^+^ T cells were first differentiated into iTreg (Foxp3+ RORgt-) and subsequently re-stimulated for four additional days with increasing concentrations of IL-6, in the presence or absence of TGF-β (**Figure 1A**). By day 8 of culture, re-stimulation with IL-6 alone resulted in detectableRORγt^+^ Treg by flow cytometry (**Supplementary Figure 1**; **Figure 1B**), whereas re-stimulation with IL-6 in combination with TGF-β led to higher frequencies of Foxp3^+^RORγt^+^ cells across all IL-6 concentrations tested (**Figure 1C**). With combined IL-6 and TGF-β conditions, the frequency of Foxp3^+^RORγt^+^ cells ranged from approximately 60% to 70% of live CD4^+^ T cells, depending on the IL-6 concentration (**Figure 1D**). In contrast, re-stimulation with IL-6 alone resulted in higher frequencies of Foxp3^-^RORγt^+^ cells, consistent with Th17 differentiation, in an IL-6 dose-dependent manner (**Figure 1F**). Based on these results, a condition combining 5 ng/mL IL-6 with 5 ng/mL TGF-β was selected for subsequent experiments, as it yielded the highest frequencies of RORγt^+^ Treg (**Figure 1D**) while concomitantly resulting in lower frequencies of conventional iTreg (**Figure 1E**), Th17 cells (**Figure 1F**), and double-negative cells (Foxp3^-^RORγt^-^) (**Figure 1G**).

**Figure 2:**
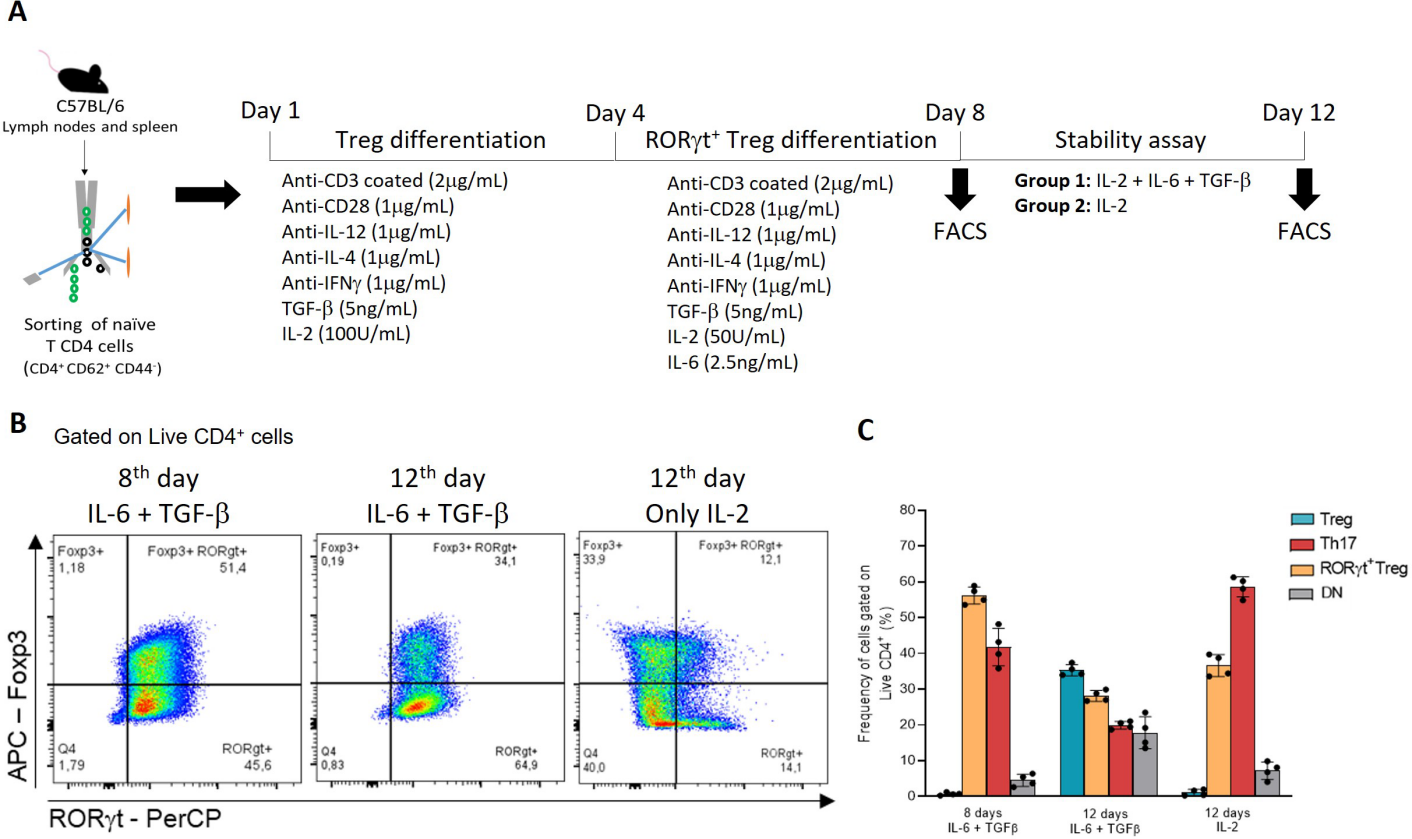
*In vitro* stability test after eight days of differentiation. **(A)** overview of RORγt^+^ Treg *in vitro* differentiation protocol followed by the stability assay. **(B)** representative gating of RORγt Treg cultured for eight and twelve days in the presence of IL-6 +TGF-β and IL-2 or IL-2 alone. **(B)** frequencies of populations analyzed by flow cytometry at each time point, obtained from a pool of mice (n=4).

To evaluate the temporal stability of the Foxp3^+^RORγt^+^ population, cells were cultured for an additional four days following the second stimulation (12 days total) (**Figure 2A**). Under continuous IL-6 and TGF-β exposure, a reduction in the frequency of Foxp3^+^RORγt^+^ cells was observed, which was mainly associated with decreased RORγt expression (**Figure 2B**). In contrast, cultures maintained with IL-2 alone exhibited a more pronounced loss of the double-positive phenotype, accompanied by reduced co-expression of Foxp3 and RORγt (**Figure 2C**). Taken together, these results show that sequential re-stimulation of iTreg with IL-6 and TGF-β efficiently enriches for RORγt^+^ Treg *in vitro* and partially preserves this phenotype over time compared to IL-2–only conditions.

### 3.2 *In vitro*-generated RORγt^+^ Treg are capable of inhibiting effector T cell proliferation

**Figure 3:**
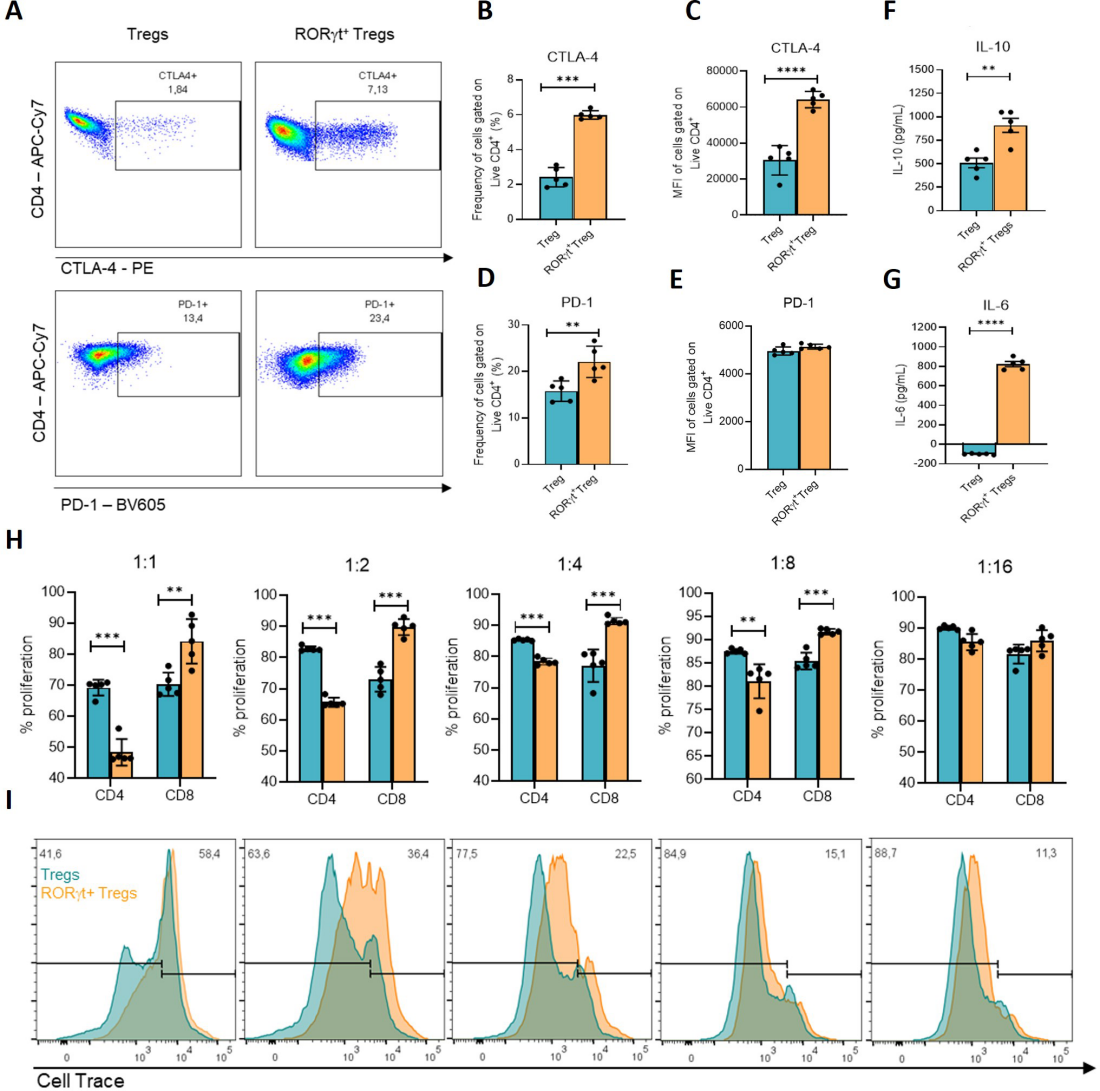
*In vitro* differentiated RORγt^+^ Treg exhibit suppressive capacity mediated by co-inhibitory molecule expression. **(A)** representative gating and quantification of CTLA-4 and PD-1 expression within live singlet CD4^+^Foxp3^+^ subsets, defined as iTreg (Foxp3^+^RORγt^-^) and RORγt^+^ Treg (Foxp3^+^RORγt^+^). **(B, C)** frequency (%) and MFI of CTLA-4 expression in iTreg and RORγt Treg. **(D, E)** frequency (%) and MFI of PD-1 expression in iTreg and RORγt Treg. **(F)** IL-10 production measured in the supernatant of iTreg and RORγt Treg cultures, analyzed by ELISA. **(G)** IL-6 production measured in the supernatant of iTreg and RORγt Treg cultures, analyzed by ELISA. **(H, G)** suppression assay of *in vitro* differentiated RORγt^+^ Treg and iTreg. Total splenocytes were labeled with CellTrace violet and plated (75 × 10³/well) in a 96-well plate. Cells sorted after *in vitro* differentiation into RORγt^+^ Treg and iTreg were co-cultured at ratios of 2:1 (150 × 10³ cells/well), 1:1, 1:2, 1:4, 1:8, and 1:16. Proliferation was stimulated with soluble anti-CD3 (1 μg/mL) and quantified by flow cytometry. Data obtained from a pool of mice (n=5). Bars represent statistical differences (*p<0.05; **p<0.01; ***p<0.001; ****p<0.0001) between groups analyzed using student’s t-test.

To assess the immune profile of *in vitro* differentiated RORγt+ Treg compared to iTreg, the expression of the co-inhibitory molecules CTLA-4 and PD-1 was evaluated by flow cytometry within Foxp3^+^ subsets following *in vitro* differentiation (**Supplementary Figure 2**; **Figure 3A**). RORγt^+^ Treg displayed higher frequencies of CTLA-4^+^ (**Figure 3B**) and PD-1^+^ (**Figure 3C**) cells than iTreg. Yet, CTLA-4 MFI (**Figure 3D**) was higher in the double-positive population, while PD-1 MFI (**Figure 3E**) showed no difference between the two Treg subtypes. Cytokine production was assessed in supernatants collected from these Treg-only cultures. RORγt^+^ Treg produced higher levels of IL-10 than iTreg (**Figure 3F**), while IL-6, a pleiotropic cytokine produced *in vivo* by RORγt+ Treg, was also detected at higher concentrations in Foxp3^+^RORγt^+^ Treg cultures (**Figure 3G**).

To assess the suppressive capacity of *in vitro*-generated RORγt^+^ Treg, suppression assays were performed using CellTrace Violet–labeled total splenocytes co-cultured with sorted iTreg or RORγt^+^ Treg at decreasing Treg-to-responder ratios (**Figure 3H**). Proliferation of CD4^+^ and CD8^+^ T cells was quantified after 72 h of stimulation. Foxp3^+^RORγt^+^ Treg reduced CD4^+^ T cell proliferation across multiple ratios when compared to iTreg (**Supplementary Figure 3**; **Figure 3I**). Collectively, these data show that *in vitro*–generated RORγt^+^ Treg display enhanced suppressive capacity, increased expression of co-inhibitory receptors, and a cytokine profile divergent from conventional iTreg.

### 3.3 Metabolic profiling of *in vitro* differentiated RORγt^+^ Treg

**Figure 4:**
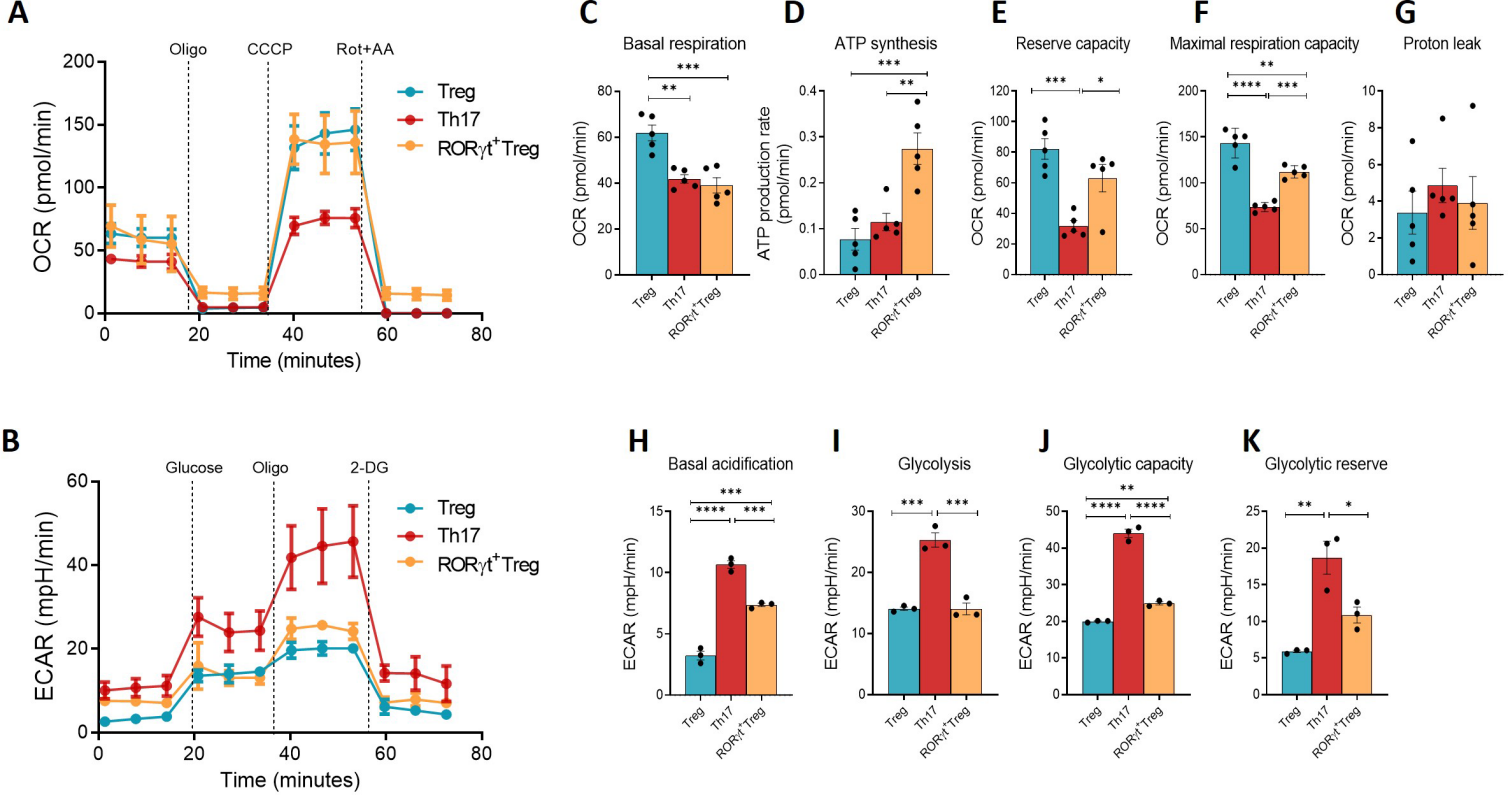
*In vitro* generated RORγt Treg are metabolically similar to iTreg. **(A)** 400,000 cells were transferred to the wells of an XF96 plate, and oxygen levels were measured before and after stimulation with oligomycin (1 μg/mL), CCCP (5 μM), and rotenone (1 μg/mL)/Antimycin-A (1 μM) (Rot+AA). **(B)** 400,000 cells were transferred to the wells of an XF96 plate, and medium acidification levels were measured before and after stimulation with glucose (25 mM), oligomycin (1 μg/mL), and 2-DG (20 mM). **(C-G)** Parameter analyses generated from the ratios between oxygen consumption rate values obtained after each stimulus (pool of mice, n=4). **(H-K)** Parameter analyses generated from the ratios between acidification rate values obtained after each stimulus (pool of mice, n=3). Bars indicate statistical significance (*p<0.05; **p<0.01; ***p<0.001; ****p<0.0001) between groups, analyzed using a one-way ANOVA test.

Metabolic activity of *in vitro*-differentiated T cell subsets was assessed using extracellular flux analysis. Naïve CD4^+^ T cells were differentiated into iTreg and Th17 cells for four days, or into RORγt^+^ Treg for eight days, and oxygen consumption rate (OCR) and extracellular acidification rate (ECAR) were measured (**Figures 4A, B**). We observed that the oxygen consumption profile and mitochondrial activity of RORγt^+^ Treg cells were very similar to those of iTreg cells, suggesting greater ATP production via OXPHOS. RORγt^+^ Treg and Treg exhibited significantly elevated basal OCR and reserve capacity compared to Th17 cells (**Figures 4C, E**). Additionally, the fraction of basal OCR involved in ATP-coupled respiration was statistically higher in RORgt+ Treg cells compared to Treg and Th17 cells (**Figures 4D–G**). As reported in the literature, Th17 cells have diminished mitochondrial activity and greater medium acidification (indicative of lactate production) compared to iTreg (**Figure 4B**). Interestingly, RORγt^+^ Treg exhibited basal medium acidification more similar to that observed in Th17 cells (**Figure 4H**). In the other glycolytic parameters, RORγt^+^ Treg were an intermediate between iTreg and Th17 cells (**Figures 4I–K**). Thus, we demonstrate that RORγt^+^ Treg cells are in a distinct metabolic state compared with Th17 cells, suggesting they are a distinct entity in terms of metabolic consumption and activation, more similar to iTreg and highly OXPHOS-dependent.

## 4 Discussion

The differentiation of Foxp3^+^RORγt^+^ regulatory T cells from naïve CD4^+^ T cells is a tightly regulated process that reflects the well-documented plasticity between regulatory and inflammatory T cell lineages. Initially, the co-expression of Foxp3 and RORγt was identified as a feature associated with early stages of both Th17 and Treg differentiation, as naïve CD4^+^ T cells can transiently express both transcription factors when stimulated *in vitro* under Th17-polarizing conditions (9). From this intermediate Foxp3^+^RORγt^+^ stage, cells may subsequently differentiate toward either a regulatory or inflammatory fate depending on the balance between TGF-β and pro-inflammatory cytokines such as IL-6 and IL-23 (10). Subsequent *in vivo* studies refined this view by demonstrating that RORγt^+^ Treg constitute a functionally relevant regulatory population, capable of producing IL-10 and suppressing effector T cell proliferation, particularly in the intestinal environment (7, 11). In parallel, other studies showed that Foxp3^+^ Treg can acquire IL-17 expression in both mice and humans (12–14), leading to the concept of “inflammatory Treg,” which retain regulatory properties whilst producing pro-inflammatory cytokines. Nevertheless, accumulating evidence supports the notion that cells coexpressing Foxp3 and RORγt do not exclusively represent transient intermediates but can constitute a stable regulatory lineage.

Our data broaden these observations by showing that the sequence of transcription factor induction is an essential determinant of lineage outcome *in vitro*. While other groups differentiated RORγt^+^ Treg cells by applying low doses of IL-6 directly to naïve CD4^+^ T cells under Treg-polarizing conditions, these approaches yielded approximately half the frequency of Foxp3^+^RORγt^+^ cells and a concomitant increase in Th17 cells compared to our protocol (1, 8). In contrast, our results support a model in which prior stabilization of Foxp3 expression followed by controlled induction of RORγt favors a purer and more stable RORγt^+^ Treg population. This sequential differentiation strategy likely limits diversion toward inflammatory Th17-like fates and may better recapitulate aspects of RORγt^+^ Treg development observed *in vivo*. One main finding of our study is that RORγt^+^ Treg cells display greater suppressive activity than iTreg cells. This was demonstrated by higher CTLA-4 expression *in vitro* and in subsequent suppression assays, in which RORγt^+^ Treg effectively inhibited the proliferation of both CD4^+^ and CD8^+^ effector T cells. These results are consistent with previous studies showing that RORγt^+^ Treg cells play a key role in modulating immune responses in the gut and other mucosal sites, helping maintain immune homeostasis.

The superior suppression capacity of RORγt^+^ Treg cells observed in our experiments may be attributed to their specific transcriptional and metabolic profiles. In T cells, metabolic programming is tightly linked to differentiation state. Activation of naïve CD4^+^ T cells under Th1, Th2, or Th17 conditions is associated with upregulation of the glucose transporter GLUT1 and increased glycolytic rates, with Th17 cells exhibiting particularly high glycolytic flux (15). In contrast, T cells activated under Treg-inducing conditions display reduced glycolysis and increased reliance on OXPHOS and fatty acid oxidation (16). Consistent with this framework, our metabolic analyses revealed that RORγt^+^ Treg exhibit a predominantly oxidative metabolic profile similar to iTreg, despite displaying intermediate glycolytic activity relative to Th17 cells. These findings match the concept that T cell metabolic pathways can influence functional outcomes, with OXPHOS associated with more regulatory, less inflammatory profiles, as seen in RORγt^+^ Treg (17). Interestingly, although RORγt^+^ Treg cells exhibited a basal level of extracellular acidification similar to that of Th17 cells, they still maintained a more mitochondrial-dominant metabolic profile, as evidenced by their higher OCR. This suggests that RORγt^+^ Treg balance glycolytic and oxidative metabolic pathways to support their immunosuppressive function while averting the excessive pro-inflammatory effects seen in Th17 cells.

Collectively, our results support the concept that RORγt^+^ Treg represent a distinct regulatory subset whose stability and function depend on both transcriptional programming and metabolic configuration. By combining sequential transcription factor induction with controlled cytokine exposure, our *in vitro* protocol allows the generation of RORγt^+^ Treg that recapitulate key functional and metabolic features described *in vivo*. This system provides a valuable experimental framework to further investigate the mechanisms controlling RORγt^+^ Treg biology and exploring their possible roles in immune-mediated diseases characterized by chronic inflammation, such as inflammatory bowel disease and cancer.

## 5 Limitations of the study

Although promising results were observed *in vitro*, the results presented here require validation *in vivo* to confirm that RORγt^+^ Treg maintain their immunoregulatory and metabolic profiles in a physiological setting. The *in vivo* immune microenvironment is much more complex and dynamic, and it is necessary to determine whether RORγt^+^ Treg cells retain the same suppressive capacity and metabolic features when transferred into animal disease models. Furthermore, the ability of these cells to modulate immune responses in various tissue environments (e.g., the gut versus other peripheral tissues) remains to be fully elucidated.

The differentiation of RORγt^+^ Treg from naïve CD4+ T cells is still a relatively new and evolving process. Although we optimized our protocol to generate a stable population of RORγt^+^ Treg, the differentiation efficiency can vary across experiments and may be affected by factors such as the purity of the starting T cell population, cytokine concentrations, and even differences in mice’s genetic backgrounds. This variation may affect the reproducibility of results and the interpretation of functional assays. To summarize, while our study provides important insights into the differentiation, function, and metabolism of RORγt^+^ Treg cells, additional *in vivo* confirmation and more comprehensive metabolic profiling of these cells are necessary to fully understand their roles in immune regulation and their potential therapeutic applications.

## Data Availability

The raw data supporting the conclusions of this article will be made available by the authors, without undue reservation.

## Supplementary material

Supplementary Table 1 Cytokines and neutralizing antibodies used for *in vitro* differentiation.

Supplementary Table 2 Flow cytometry staining dyes and antibodies.

Supplementary Table 3 Flow cytometry staining dyes and antibodies.

Supplementary Figure 1 Flow cytometry gating strategy for identification of RORγt^+^ Treg after *in vitro* differentiation. Naïve CD4^+^ T cells were differentiated *in vitro* under RORγt^+^ Treg–polarizing conditions. Cells were stained with Live/Dead Viability Dye, anti-CD4, anti-Foxp3, and anti-RORγt. Representative plots show the sequential gating strategy. Lymphocytes were gated based on FSC and SSC, followed by exclusion of doublets using FSC-A versus FSC-H and selection of viable cells (Live/Dead^-^). CD4^+^ T cells were used as the parent population for subsequent analyses. Foxp3 and RORγt expression was assessed within live singlet CD4^+^ cells, allowing the identification of Foxp3^+^RORγt^+^, Foxp3^+^RORγt^-^, Foxp3^-^RORγt^+^, and double-negative populations. Frequencies are expressed as percentages of live CD4^+^ T cells. Gates were defined using single-stained and unstained controls for Foxp3 and RORγt. A minimum of 100,000 total events was acquired per sample.

Supplementary Figure 2 Gating strategy for CTLA-4 and PD-1 expression in differentiated CD4^+^ T cell subsets. *In vitro*–differentiated cells were stained with Live/Dead Viability Dye, anti-CD4, anti-Foxp3, anti-RORγt, anti-CTLA-4 and anti-PD-1. After gating on lymphocytes, singlets, and viable cells, CD4^+^ T cells were selected as the parent gate. Foxp3 and RORγt expression was used to define Treg and RORγt^+^ Treg subsets. CTLA-4 and PD-1 expression was subsequently quantified within the indicated parent populations. Data are presented as the percentage of positive cells within each gate, and for CTLA-4 and PD-1 the MFI was quantified too. Gates were set using single-stained and unstained controls for all markers. At least 100,000 total events were collected per sample prior to analysis.

Supplementary Figure 3 Flow cytometry gating strategy for suppression assay. Splenocytes were labeled with Cell Tracer dye and analyzed by flow cytometry after co-culture. Representative plots illustrate the gating strategy. Lymphocytes were identified by FSC and SSC, followed by singlet selection. CD4^+^ and CD8^+^ T cell populations were gated as indicated, and Cell Tracer dilution was analyzed within the responder cell gate. Proliferating cells were defined based on reduced Cell Tracer fluorescence, whereas non-proliferating cells retained high fluorescence intensity. Frequencies are expressed as percentages of total gated responder cells. Gates were established using unstimulated, single-stained and unstained controls. A minimum of 100,000 total events was acquired per sample.
