# Supplementary material for: In vitro generation of RORγt+ regulatory T cells reveals enhanced immunosuppressive function and OXPHOS-dependent metabolism
Source: Front Immunol. 2026 May 21;17:1742866. doi: 10.3389/fimmu.2026.1742866 (PMC13233403; doi:10.3389/fimmu.2026.1742866)
Supplement: Supplementary Table 1 — Cytokines and neutralizing antibodies used for in vitro differentiation. [file Table1.docx]

**Table S1.** Cytokines and neutralizing antibodies used for *in vitro* differentiation.

| *Cytokine/Antibody* | *Use concentration* | *Company* | *Antibody clone* | *Catalog number* |
| --- | --- | --- | --- | --- |
| Anti-CD3 | 2µg/mL | BioLegend | 17A2 | 100202 |
| Anti-CD28 | 1µg/mL | BioLegend | 37.51 | 117004 |
| Anti-IL-4 | 1µg/mL | BD Pharmingen | 11B11 | 554432 |
| Anti-IFNγ | 1µg/mL | BD Pharmingen | XMG1.2 | 554408 |
| Anti-IL-12/IL-23 p40 | 1µg/mL | BioLegend | C17.8 | 505202 |
| rmIL-2 | 100U/mL or 50U/mL | R&D Systems | - | 402-ML |
| rmTGF-β | 5ng/mL | R&D Systems | - | 7666-MB |
| rmIL-6 | 2,5-5ng/mL | Peprotech | - | 1:100 |
