# Supplementary material for: In vitro generation of RORγt+ regulatory T cells reveals enhanced immunosuppressive function and OXPHOS-dependent metabolism
Source: Front Immunol. 2026 May 21;17:1742866. doi: 10.3389/fimmu.2026.1742866 (PMC13233403; doi:10.3389/fimmu.2026.1742866)
Supplement: Supplementary Table 2 — Flow cytometry staining dyes and antibodies. [file Table2.docx]

**Table S2.** Flow cytometry staining dyes and antibodies.

| *Staining* | *Fluorophore* | *Company* | *Catalog number* | *Dilution* |
| --- | --- | --- | --- | --- |
| Viability | Live\dead fixable aqua | Invitrogen | L34966 | 1:1000 |
| anti-CD4 | APC/Cy7 | BioLegend | 100526 | 1:200 |
| Anti-CTLA-4 | PE | BD Pharmingen | 553720 | 1:200 |
| Anti-PD-1 | BV605 | BD Pharmingen | 563059 | 1:200 |
| anti-RORγt | PerCP-Cy5.5 or BV421 | BD Pharmingen | 562894 | 1:100 |
| anti-Foxp3 | APC or FITC | BioLegend | 560401 | 1:100 |
