# Supplementary material for: In vitro generation of RORγt+ regulatory T cells reveals enhanced immunosuppressive function and OXPHOS-dependent metabolism
Source: Front Immunol. 2026 May 21;17:1742866. doi: 10.3389/fimmu.2026.1742866 (PMC13233403; doi:10.3389/fimmu.2026.1742866)
Supplement: Supplementary Table 3 — Flow cytometry staining dyes and antibodies. [file Table3.docx]

**Table S3.** Flow cytometry staining dyes and antibodies.

| *Staining* | *Fluorophore* | *Company* | *Catalog number* | *Dilution* |
| --- | --- | --- | --- | --- |
| Viability | Live\dead fixable aqua | Invitrogen | L34966 | 1:1000 |
| Proliferation | Cell Trace Violet | Invitrogen | C34557 | - |
| anti-CD4 | APC/Cy7 | BioLegend | 100526 | 1:200 |
| Anti-CD8 | FITC | BioLegend | 100706 | 1:200 |
