# Supplementary figures and images for: In vitro generation of RORγt+ regulatory T cells reveals enhanced immunosuppressive function and OXPHOS-dependent metabolism
Source: Front Immunol. 2026 May 21;17:1742866. doi: 10.3389/fimmu.2026.1742866 (PMC13233403; doi:10.3389/fimmu.2026.1742866)

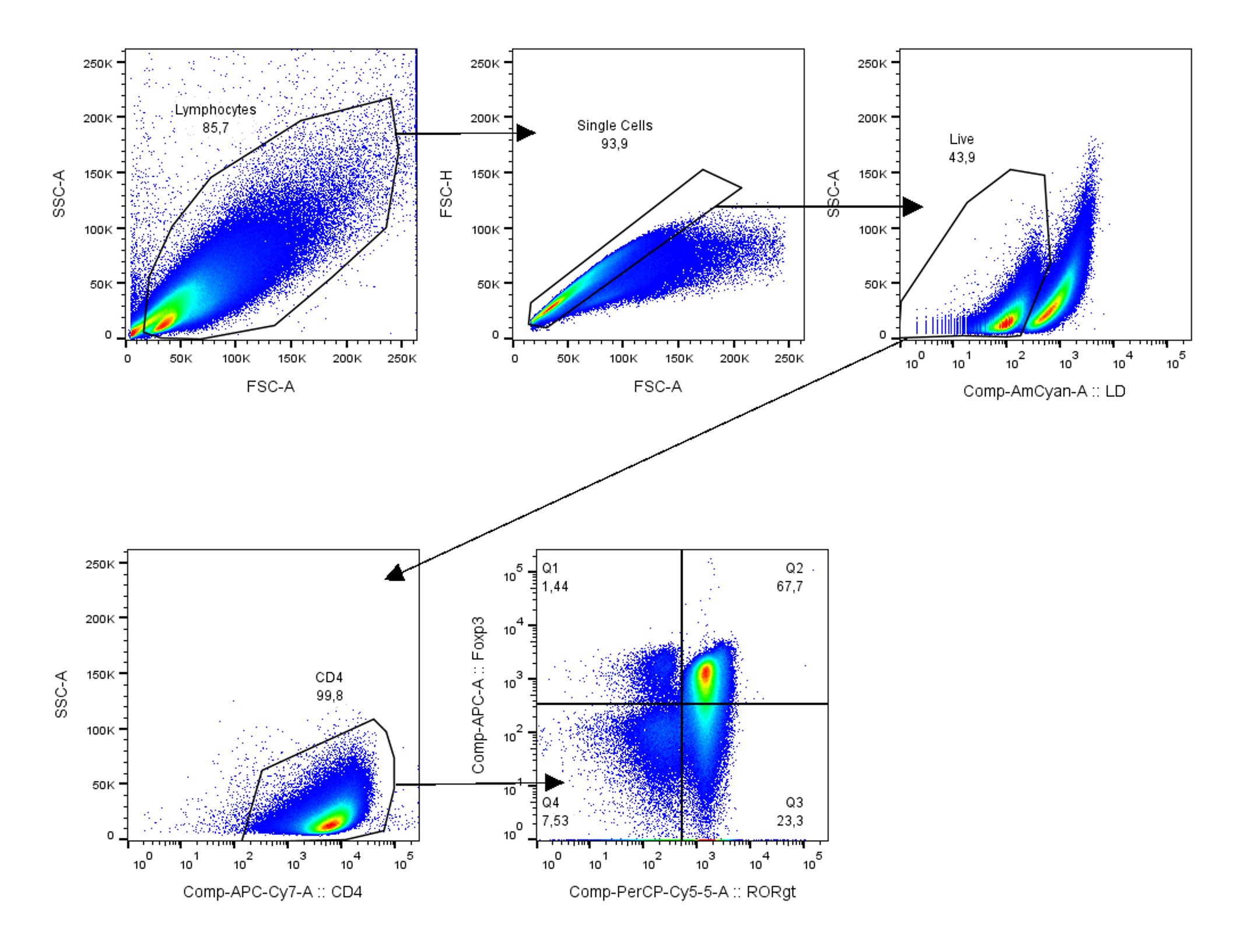

Supplement: Supplementary Figure 1 — Flow cytometry gating strategy for identification of RORγt+ Treg after in vitro differentiation. Naïve CD4+ T cells were differentiated in vitro under RORγt+ Treg–polarizing conditions. Cells were stained with Live/Dead Viability Dye, anti-CD4, anti-Foxp3, and anti-RORγt. Representative plots show the sequential gating strategy. Lymphocytes were gated based on FSC and SSC, followed by exclusion of doublets using FSC-A versus FSC-H and selection of viable cells (Live/Dead-). CD4+ T cells were used as the parent population for subsequent analyses. Foxp3 and RORγt expression was assessed within live singlet CD4+ cells, allowing the identification of Foxp3+RORγt+, Foxp3+RORγt-, Foxp3-RORγt+, and double-negative populations. Frequencies are expressed as percentages of live CD4+ T cells. Gates were defined using single-stained and unstained controls for Foxp3 and RORγt. A minimum of 100,000 total events was acquired per sample. [file Image1.jpeg]

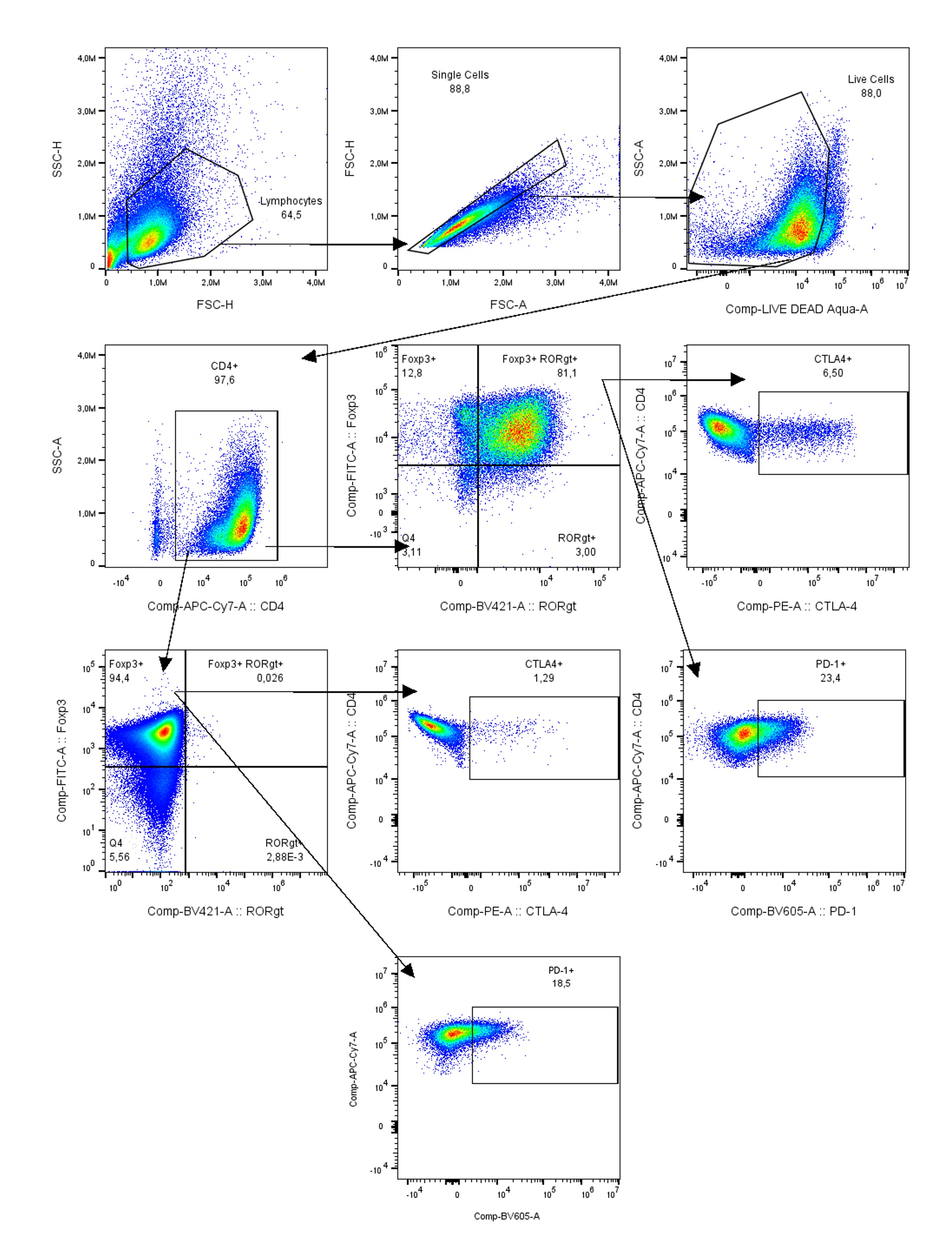

Supplement: Supplementary Figure 2 — Gating strategy for CTLA-4 and PD-1 expression in differentiated CD4+ T cell subsets. In vitro–differentiated cells were stained with Live/Dead Viability Dye, anti-CD4, anti-Foxp3, anti-RORγt, anti-CTLA-4 and anti-PD-1. After gating on lymphocytes, singlets, and viable cells, CD4+ T cells were selected as the parent gate. Foxp3 and RORγt expression was used to define Treg and RORγt+ Treg subsets. CTLA-4 and PD-1 expression was subsequently quantified within the indicated parent populations. Data are presented as the percentage of positive cells within each gate, and for CTLA-4 and PD-1 the MFI was quantified too. Gates were set using single-stained and unstained controls for all markers. At least 100,000 total events were collected per sample prior to analysis. [file Image2.jpeg]

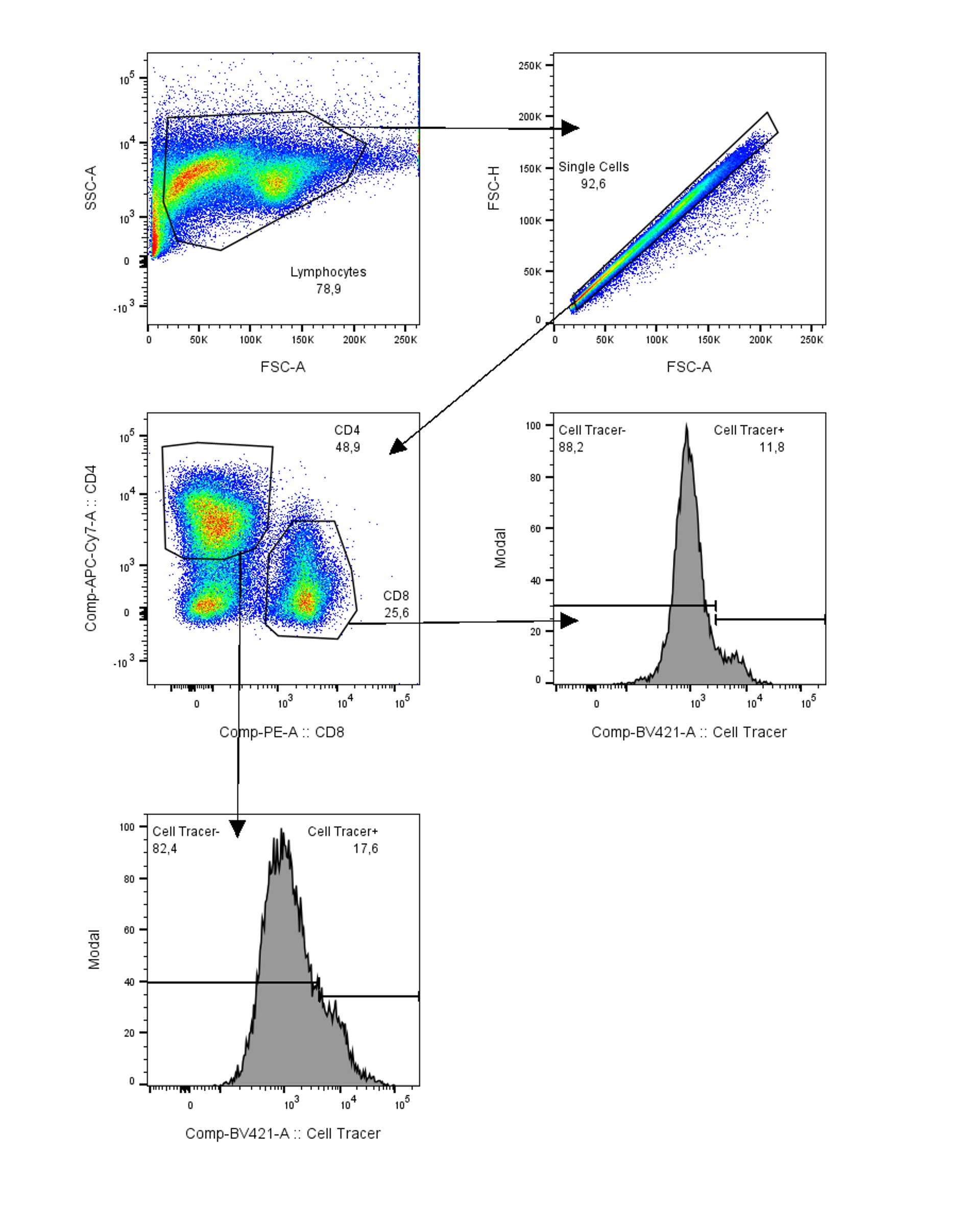

Supplement: Supplementary Figure 3 — Flow cytometry gating strategy for suppression assay. Splenocytes were labeled with Cell Tracer dye and analyzed by flow cytometry after co-culture. Representative plots illustrate the gating strategy. Lymphocytes were identified by FSC and SSC, followed by singlet selection. CD4+ and CD8+ T cell populations were gated as indicated, and Cell Tracer dilution was analyzed within the responder cell gate. Proliferating cells were defined based on reduced Cell Tracer fluorescence, whereas non-proliferating cells retained high fluorescence intensity. Frequencies are expressed as percentages of total gated responder cells. Gates were established using unstimulated, single-stained and unstained controls. A minimum of 100,000 total events was acquired per sample. [file Image3.jpeg]
